# Evaluation of the Broad-Range PCR-Electrospray Ionization Mass Spectrometry (PCR/ESI-MS) System and Virus Microarrays for Virus Detection

**DOI:** 10.3390/v6051876

**Published:** 2014-04-25

**Authors:** Lanyn P. Taliaferro, Teresa A. Galvin, Hailun Ma, Syed Shaheduzzaman, Dhanya K. Williams, Dustin R. Glasner, Arifa S. Khan

**Affiliations:** Laboratory of Retroviruses, Division of Viral Products, Center for Biologics Evaluation and Research, U.S. Food and Drug Administration, 8800 Rockville Pike, HFM-454, Bethesda, MD 20892, USA; E-Mails: lanyn.taliaferro@fda.hhs.gov (L.P.T.); teresa.galvin@fda.hhs.gov (T.A.G.); hailun.ma@fda.hhs.gov (H.M.); syed.shaheduzzaman@fda.hhs.gov (S.S.); dhanya.williams@fda.hhs.gov (D.K.W.)

**Keywords:** broad-range PCR amplification, electrospray ionization-mass spectrometry, PLEX-ID, virus microarray, RT-PCR, endogenous retroviruses

## Abstract

Advanced nucleic acid-based technologies are powerful research tools for novel virus discovery but need to be standardized for broader applications such as virus detection in biological products and clinical samples. We have used well-characterized retrovirus stocks to evaluate the limit of detection (LOD) for broad-range PCR with electrospray ionization mass spectrometry (PCR/ESI-MS or PLEX-ID), RT-PCR assays, and virus microarrays. The results indicated that in the absence of background cellular nucleic acids, PLEX-ID and RT-PCR had a similar LOD for xenotropic murine retrovirus-related virus (XMRV; 3.12 particles per µL) whereas sensitivity of virus detection was 10-fold greater using virus microarrays. When virus was spiked into a background of cellular nucleic acids, the LOD using PLEX-ID remained the same, whereas virus detection by RT-PCR was 10-fold less sensitive, and no virus could be detected by microarrays. Expected endogenous retrovirus (ERV) sequences were detected in cell lines tested and known species-specific viral sequences were detected in bovine serum and porcine trypsin. A follow-up strategy was developed using PCR amplification, nucleotide sequencing, and bioinformatics to demonstrate that an RD114-like retrovirus sequence that was detected by PLEX-ID in canine cell lines (Madin-Darby canine kidney (MDCK) and Cf2Th canine thymus) was due to defective, endogenous gammaretrovirus-related sequences.

## 1. Introduction

Advanced nucleic acid-based technologies such as massively parallel or deep sequencing (MPS), broad-range PCR with electrospray ionization mass spectrometry (PCR/ESI-MS; PLEX-ID platform), and virus microarrays have been used for research investigations of a variety of clinical samples, environmental materials, and some biological products, thus demonstrating their capabilities for broad virus detection and novel virus discovery [1,2,3,4,5]. Additionally, viral sequences have been identified in various biological raw materials used for cell culture such as bovine serum [6] and porcine trypsin [7]. Detection of latent viruses [8,9] or novel viruses [10] has also been reported in some cell lines by using the new methods. The development of new vaccines for current and re-emerging diseases such as AIDS and pandemic influenza has necessitated the use of novel cell substrates of human, canine, avian, and insect origin. A major challenge regarding use of these cell lines for vaccine manufacture has been addressing regulatory safety concerns related to the potential presence of viruses that may not be detected by the routinely recommended virus detection assays for cell line safety such as latent DNA viruses and endogenous retroviruses (ERVs) as well as occult and unknown viruses [11]. While these technologies exhibit the potential for characterization of biological materials, their regulatory application requires rigorous and systematic evaluation including assay standardization and development of follow-up strategies for determining the biological relevance and significance of a positive result. 

Conventional PCR assays are highly sensitive but rely on specific primers, thus limiting detection to known virus sequences. Virus detection using cell-based assays is generally based upon virus replication and the ability to produce a visual effect such as cytopathicity or hemadsorption/hemagglutination [11]; therefore, latent viruses and viruses lacking such biological properties may not be detected [12]. Accordingly, chemical induction assays can be used to activate latent viruses to enhance their detection [13]; however, the induced viruses may be novel and not detected by the currently available assays. For this reason, it is important to evaluate new technologies such as PLEX-ID, virus arrays, and MPS for broad virus detection. 

The PLEX-ID platform amplifies nucleic acids using broad-range PCR primers followed by electrospray mass-spectrometry to detect a variety of microbial agents, such as bacteria, fungi, mycoplasma and viruses [14,15,16]. Conserved regions are used to design broad-range primers for detection of virus families. The weight of the amplicons is determined by mass spectrometry and a base count (BC) composition of A, G, C, and T is generated; hence, known or novel organisms can be detected and identified by comparing genomic signatures to a curated BC database [15,17,18,19]. In the case of unknown microbial agents, a genomic signature is generated and the target family is identified [10,15]. Currently, the PLEX-ID Biopharma viral assays consists of four panels, which as a set, can detect approximately 35 viral families and potentially more than 1000 species belonging to the targeted viral families [20]. Although the number of samples analyzed per set is limited, the PLEX-ID platform can run and generate data for a large number of plates in one run. 

Virus microarrays consist of oligomers or probes designed based upon conserved and specific sequences across multiple regions of known viruses and/or bacteria. DNA or cDNA is fluorescently labeled and hybridized to the arrays. A scanner detects fluorescence if the sample DNA or cDNA binds a probe and the data is analyzed by customized software. The Lawrence Livermore microbial detection array version 2 (LLMDA v.2) can detect 2000 viruses as well as 900 bacteria [4], and the ViroChip v.5 (University of California, San Francisco, CA, USA) can detect ~2000 viruses [2,21,22]. 

In this study, we have compared the PLEX-ID, virus microarrays, and reverse transcriptase (RT)-PCR assays for detection of known viruses and have used the PLEX-ID for evaluation of various vaccine-related cell lines. Furthermore, we have developed a follow-up strategy for identification of a positive result detected by the PLEX-ID to determine the relevance and significance of the nucleic acid detection signal for safety evaluation in biologics. 

## 2. Results and Discussion

### 2.1. Limit of Virus Detection

Well-characterized retrovirus stocks of xenotropic murine retrovirus-related virus (XMRV) and simian foamy virus serotype 1 (SFV-1; recently designated as SFVmcy-1 [23]), with known infectious titer and number of RT-containing particles, were used to create RNA dilution panels to determine the LOD of these viruses using PLEX-ID, RT-PCR assays, and virus microarrays. Sf9 cells were chosen to provide background nucleic acids for virus spiking studies since insect viruses were not detected by any of these assays (discussed below; Section 2.2.1). Although the LLMDA v.5 arrays could have detected insect errantiviruses, the generally used bioinformatics algorithm excluded detection of these sequences (discussed below; Section 2.2.2). 

#### 2.1.1. RT-PCR Assays

**Figure 1:**
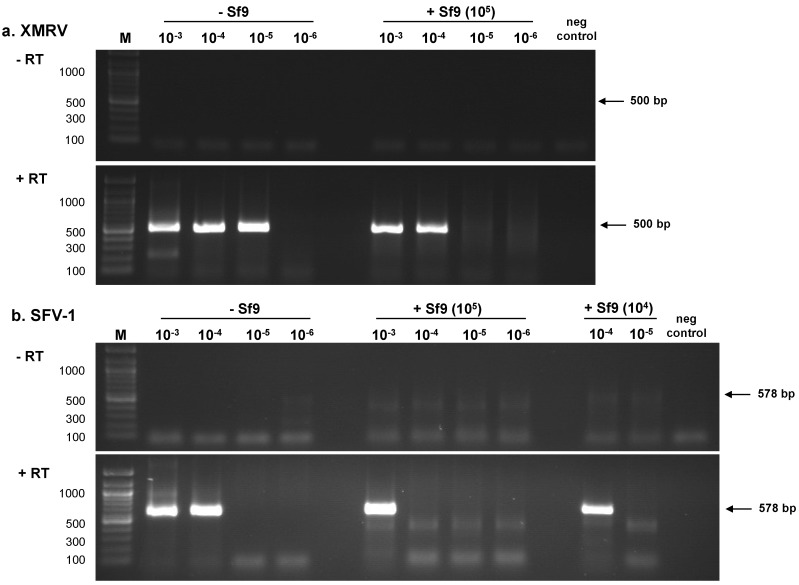
Determination of retrovirus LOD using RT-PCR assays. Total RNA was extracted from each virus dilution to create XMRV and SFV-1 RNA panels (10^0^–10^−7^) in the absence and presence of 10^5^ or 10^4^ cell equivalents of Sf9 total nucleic acids as described in Materials and Methods. A subset of the RNA panels (10^−3^–10^−6^) was subjected to nested RT-PCR assays. An RT minus (−RT) control of the samples shows the absence of residual cellular DNA and the negative, no template control for the PCR shows the absence of contamination in the assay. (**a**) XMRV *gag* primers; (**b**) SFV-1 *gag* primers. The size of fragments in the 100 bp marker (M) is indicated in base pairs (bp).

Initially, the entire dilution series of XMRV (10^0^–10^−7^) and SFV-1 (10^0^–10^−7^) were tested (5 µL sample) in the absence of background nucleic acids by RT-PCR assays to determine sensitivity of virus detection. Based upon the results, a subset of virus dilutions (10^−3^–10^−7^ for XMRV and 10^−3^–10^−6^ for SFV-1) were tested three times by RT-PCR assays in a background of water and in 10^5^ or 10^4 ^cell equivalents of Sf9 nucleic acids: the results of the LOD from the three assays were similar (one is shown in Figure 1). Complementary DNA (cDNA) synthesized from the virus dilution panels was subjected to nested PCR amplification using XMRV *gag* primers or SFV-1 *gag* primers (Figure 1, a and b, respectively; +RT panels). An RT-minus PCR control (−RT panels) was negative indicating the absence of cellular DNA contamination in the RNA preparations. The LOD in the absence of Sf9 nucleic acids for XMRV was 3.12 particles per µL (Table 1) and for SFV-1 was 40.2 particles per µL (Table 2); however, in both cases virus detection was 10-fold less sensitive in the presence of 10^5^ cell equivalents of Sf9 nucleic acids. It is noteworthy that the sensitivity of SFV-1 detection in a background of 10^4^ cell equivalents of Sf9 nucleic acids was similar to virus detection in the absence of Sf9 nucleic acids. (Table 2; not done for XMRV).

**Table 1:** Limit of detection using XMRV RNA panel.

Sample			PLEX-ID		ViroChip		RT-PCR ^a^		
RNA Dilution ^b^	TCID_50_/mL	Particles/µL ^c^	(-)Sf9	+10^5^	(-)Sf9	+10^5^	(-)Sf9	(-)Sf9	+10^5^
10^−3^	10^1.5^	312	+	+	+	nt ^d^	nt	+	+
10^−4^	10^0.5^	31.2	+	+	+	nt	+	+	+
10^−5^	10^−0.5^	3.12	+	+	+	-	+	+	-
10^−6^	10^−1.5^	0.312	-	-	+	-	+	-	-
10^−7^	10^−2.5^	0.0312	-	-	-	nt	-	-	-

^a^ Results from nested PCR with XMRV *gag* primers; ^b^ Viral RNA dilution series were spiked into RNase/DNase free water [(−)Sf9] or present (+) in 10^5^ or 10^4^ cell equivalents of Sf9 total nucleic acids; ^c^ Calculated based upon RT activity determined by TSF-PERT assay; ^d^ Not tested.

**Table 2:** Limit of detection using SFV-1 RNA panel.

Sample			PLEX-ID	LLMDA		RT-PCR ^a^		
RNA Dilution ^b^	TCID_50_/mL	Particles/µL ^c^	(-)Sf9	(-)Sf9	+10^4^	(-)Sf9	+10^5 ^	+10^4^
10^−3^	10^2.5^	402	nt ^d^	nt	nt	+	+	nt
10^−4^	10^1.5^	40.2	-	+	nt	+	-	+
10^−5^	10^0.5^	4.02	-	+	-	-	-	-
10^−6^	10^−0.5^	0.402	-	-	-	-	-	nt

^a^ Results from nested PCR with SFV-1 *gag* primers; ^b^ Viral RNA dilution series were spiked into RNase/DNase free water [(−)Sf9] or present (+) in 10^5^ or 10^4^ cell equivalents of Sf9 total nucleic acids; ^c^ Calculated based upon RT activity determined by TSF-PERT assay; ^d^ Not tested.

#### 2.1.2. PLEX-ID and Virus Arrays

Based upon the RT-PCR results, selected virus RNA samples were tested by PLEX-ID and virus arrays for comparative LOD analysis. Most samples were tested at least two times and a number of samples were independently tested in our laboratory using an on-site PLEX-ID machine and beta-test panels (data not shown), and at Athogen (Athogen, Ibis Biosciences, Carlsbad, CA, USA) using updated Biopharm Viral Assays [20]: similar results were obtained. The results obtained using the PLEX-ID and virus arrays to analyze the XMRV RNA panel and the SFV-1 RNA panel are shown in Table 1 and Table 2, respectively. The results of the RT-PCR assays shown in Figure 1 are also included in the tables for comparison. PLEX-ID detected XMRV in the RNA dilution of 10^−5^ both in the absence and presence of Sf9 total nucleic acids, which corresponded to 3.12 particles per µL. SFV-1 was not detected due to the absence of virus family-specific primers in the PLEX-ID panels. 

At a quantile threshold of 0.99 [4], LLMDA could detect the 10^−6^ XMRV RNA dilution in water, which corresponded to 0.312 virus particles per µL; however, no signal was seen in the background of 10^5^ or 10^4^ cell equivalents of Sf9 total nucleic acids. To further evaluate the specificity of the XMRV signal detected with the 10^−6 ^RNA dilution, filtered supernatant from uninfected LNCaP cells and the complete medium used for virus propagation were tested by LLMDA; negative results were obtained (data not shown). LLMDA detected SFV-1 in the 10^−5^ RNA dilution, which corresponded to an LOD of 4.02 virus particles per µL. 

Analysis of XMRV using the ViroChip indicated an LOD of 0.312 particles per µL (RNA dilution 10^−6^) in the absence of Sf9 nucleic acids. Only viral sequences with a threshold of at least five or more probes matching the viral genome with 94%–100% identity were reported. Samples in the presence of Sf9 total nucleic acids were not tested using the ViroChip. It should be noted that virus detection in the absence of Sf9 nucleic acids by LLMDA was a log more sensitive than PLEX-ID and RT-PCR; however, there was no virus detection using LLMDA in the background of 10^5^ or 10^4^ cell equivalents of Sf9 total nucleic acids. PLEX-ID had the same LOD in the absence and presence of 10^5^ cell equivalents of Sf9 total nucleic acids and RT-PCR had a similar LOD in the absence and presence of 10^4^ cell equivalents of Sf9 total nucleic acids. 

### 2.2. Investigation of Cell Lines and Cell Culture Reagents

#### 2.2.1. PLEX-ID Analysis

A variety of cell lines, used in research and relevant to biological products, were evaluated for virus detection by the PLEX-ID since this platform was sensitive even in the presence of background cellular nucleic acids. Total nucleic acids were extracted from cell pellets and analyzed using PLEX-ID Biopharma Viral Assay Kits (Athogen, Ibis Biosciences, Carlsbad, CA, USA). In addition, biological raw materials such as complete medium, FBS, trypsin, PBS, and ultra-Pure dH_2_O were also tested by PLEX-ID (data not shown). Bovine viral diarrhea virus 1 (BVDV-1) sequences were detected in complete medium and in FBS [6]. Porcine circovirus type 2 (PCV-2) sequences were detected in trypsin, which was expected since this virus is ubiquitous in swine [24]. PBS and ultra-pure dH_2_O were free of detectable viral sequences.

The cell lines analyzed in this study and the PLEX-ID results are shown in Table 3. Several endogenous retroviruses belonging to alpharetrovirus, betaretrovirus, or gammaretrovirus genera were identified [25,26]. Avian leukosis virus (ALV) sequences were detected by PLEX-ID in all human cell lines except HeLa. Furthermore, in-depth mass spectrometry analysis of each well, revealed “unknown” BCs that were not present in Athogen’s curated database. Some of the peaks were found to be potential “unidentified” alpharetrovirus sequences, which were compared to Athogen’s curated database. The analysis revealed that the BCs for the unidentified alpharetrovirus sequences were near-matches to ALV, human endogenous retrovirus K (HERV-K), or human mammary tumor virus (HMTV). This information revealed that the HeLa cell line, which appeared to be free of ALV sequences, did in fact harbor ALV-like sequences with a BC three mutations away from known ALV BCs. In addition, Rous-associated virus 2 (RAV-2)-like sequences were detected in Raji, A204, and HeLa cells. Similarly, unidentified betaretrovirus sequences were detected in all of the human cell lines (Table 3) and the BCs were found to be closely related to known HERV-K BCs. This analysis revealed that A204 and HEK293 were not outliers, but simply harbored an endogenous betaretrovirus whose BC was represented in the curated database, whereas the other human cell lines harbored variants of an endogenous betaretrovirus not found in the curated database. In addition, simian retrovirus 1 (SRV-1)-related sequences were detected in A204 and HEK293. Since alpharetroviruses and betaretroviruses were found in all of the human cell lines, a BLAST search was performed using their respective PLEX-ID forward and reverse primer sequences (data not shown). The analysis revealed multiple hits to human chromosomes, suggesting that the alpharetrovirus and betaretrovirus sequences detected were due to endogenous retroviral sequences known to be present in human DNA [26].

Simian cell lines were also examined using the PLEX-ID platform. Table 3 shows that SRV-1 and baboon endogenous virus (BaEV) sequences were detected in VERO. SRV-related sequences as well as BaEV-related sequences have previously been shown in VERO cells to be due to simian endogenous retroviruses (SERV) [9,27]. No unidentified BCs were found after in-depth mass spectrometry analysis. It should be noted that the SRV-1-related sequences found in the human cell lines and monkey cell lines had closely related BCs, but were not identical.

**Table 3:** PLEX-ID analysis of cell lines.

Cell Lines	Alpharetrovirus-related			Betaretrovirus-related		Gammaretrovirus-related		
	ALV	RAV-2	Unidentified	SRV-1	Unidentified	RD114	BaEV	Undistinguishable
*Human*								
A549	+ ^b^		+		+			
Raji	+	+	+		+			
MRC-5	+		+		+			
A204	+	+	+	+	+			
HEK293	+			+	+			
293T/17 ^a^	+		+		+			
HeLa		+	+		+			
*Simian*								
VERO				+			+	
CV-1				+			+	
*Canine*								
MDCK						+		+
Cf2Th						+		+

^a^ Simian virus 40 (SV40) is constitutively expressed in 293T/17 cells and was detected, but not listed in the table; ^b^ +, detection of positive signal, or a blank space is left in the absence of a signal.

PLEX-ID analysis of the canine MDCK and Cf2Th cell lines identified RD114-like gammaretrovirus sequences. Additionally, “undistinguishable” gammaretrovirus sequences were found in both cell lines whose BC matched several known gammaretroviruses (Table 3). Since the identification of RD114-like sequences in the canine cell lines was unexpected, a follow-up strategy was developed to understand the origin of this viral hit (Section 2.3). 

In addition to endogenous retroviruses, simian virus 40 (SV40) sequences were detected by PLEX-ID in 293T/17 cells, which constitutively expresses the large T antigen (footnote, Table 3) [28]. However, other viral sequences known to be present in the test cell lines were not detected; these included: adenovirus sequences in 293T/17 and HEK293 cells [29], Epstein-Barr virus (EBV) sequences in Raji cells [30] and human papillomavirus (HPV) sequences in HeLa cells [31]. Several insect cell lines (SL2, Sf9, and Hi Five), which contain insect ERVs or errantiviruses [32], were also examined but no viral sequences were detected (data not shown). Moreover, a novel rhabdovirus that was reported in insect Sf9 cells [33] was not detected by PLEX-ID even though broad-range primers designed to detect members of the Rhabdoviridae family were included in the PLEX-ID Biopharma Porcine origin viral assay used to screen the cell lines.

#### 2.2.2. Virus Arrays

A subset of cell lines tested by PLEX-ID (Raji, MDCK, VERO, Sf9, and SL2) was also analyzed using virus arrays of which VERO and Sf9 were tested at least two times. At a quantile threshold of 0.99, LLMDA detected HERV-K in VERO and Raji cells but other sequences detected by PLEX-ID such as SERV and BaEV-related in VERO and alpharetrovirus sequences (ALV- and RAV-related) in Raji cells were not detected. Furthermore, LLMDA did not detect RD114 sequences in MDCK cells that were found by PLEX-ID. Conversely, EBV was detected in Raji cells by LLMDA, but not by PLEX-ID. No viruses were detected in insect Sf9 and SL2 cell lines by PLEX-ID, however, the updated LLMDA v.5 was able to detect the nine known errantiviruses in Sf9 cells [32] and multiple ones in SL2 cells (data not shown) using a modified bioinformatics analysis and a specialized retroelement database designed by LLMDA [4]. However, a novel rhabdovirus that was found in the Sf9 cell line [33] was not detected by LLMDA.

MDCK and VERO were also tested using the ViroChip (data not shown): BaEV- and Mason-Pfizer monkey virus (MPMV)-related sequences, which are related to SRV-1, were detected in VERO; RD114 was not detected in MDCK cells. 

### 2.3. Follow-Up Strategy for a Positive Hit by PLEX-ID

The detection of a positive signal in cells using the nucleic acid-based technologies can be due to an exogenous virus infection or endogenous retroviral sequences that may be expressed as infectious or defective particles. Therefore, it is important to distinguish the origin and nature of the signal.

#### 2.3.1. PCR Amplification and Nucleotide Sequencing

**Figure 2:**
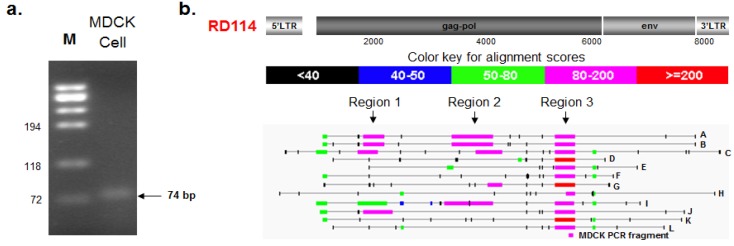
Follow up analysis of the PLEX-ID RD114 signal. (**a**) A 74 bp RD114-like gammaretrovirus fragment was amplified from total nucleic acids extracted from MDCK cells, as described in materials and methods; (**b**) Blastn analysis was conducted using the 74 bp fragment nucleotide sequence as the query against the *Canis lupus familiaris* genome database (TAXID: 9615) and several BAC clones were identified. An alignment highlighting different areas of similarity between RD114, several chromosomal *Canis lupus familiaris* BAC clones (designated A–L; see Table 4 for details), and the MDCK PCR fragment are shown.

**Figure 3:**
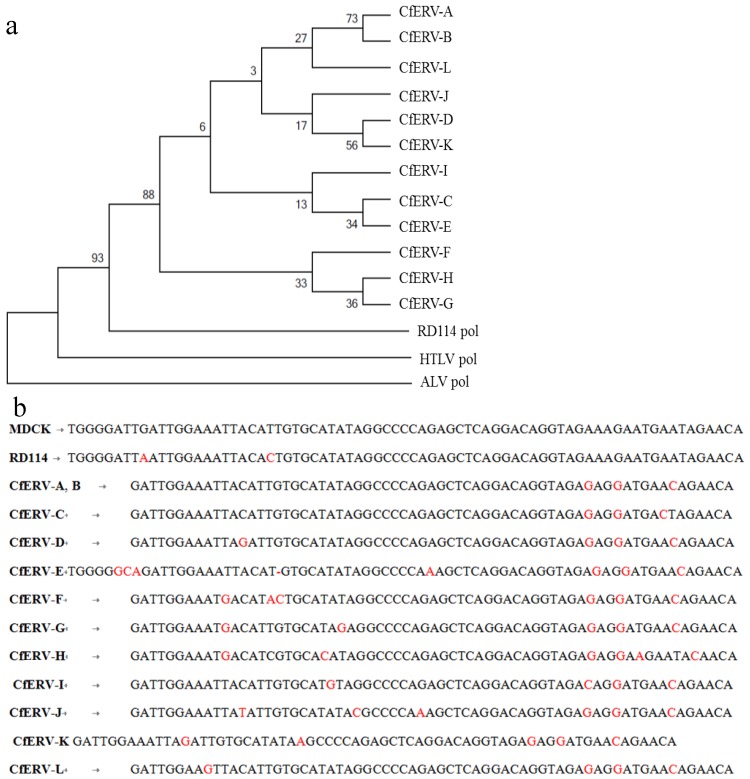
Molecular phylogenetic analysis and nucleotide sequence comparison of RD114-like CfERVs *pol* region 3. (**a**) A maximum likelihood tree based on the Kimura 2-parameter model was constructed using a conserved region of the *pol* region from BAC clones A–L (see Figure 2b) and distant homologs, HTLV-1 and ALV [34]. The bootstrap consensus tree inferred from 1000 replicates [35]. The percentage of replicate trees in which the associated taxa clustered together in the bootstrap test (1000 replicates) is shown next to the branches [35]. The analysis involved 15 nucleotide sequences. Codon positions included were 1st + 2nd + 3rd + Noncoding. All positions containing gaps and missing data were eliminated. There were a total of 134 positions in the final data set. Sequence MUSCLE alignment and evolutionary analyses were conducted in MEGA5 [36]; (**b**) Nucleotide sequence comparison of the MDCK74 sequence with the analogous region in RD114 virus and CfERVs is shown. Nucleotide differences are indicated in red.

A follow-up strategy was developed to confirm and identify RD114 retroviral sequences detected by PLEX-ID in MDCK and Cf2Th cells. In an effort to determine the origin of the PLEX-ID RD114 signal we used PCR primers that targeted the same highly conserved region of the gammaretrovirus *pol* gene as in the PLEX-ID analysis. Total nucleic acids extracted from MDCK cells were subjected to a nested PCR assay as described in materials and methods. Since the first round of PCR amplified a slightly longer fragment from the MDCK cells than the nested PCR, we used the 74 bp fragment instead of the 64 bp fragment for further analysis (Figure 2a). The DNA fragment, designated as MDCK74, was extracted by gel purification and nucleotide sequences determined. Comparison with the RD114 virus genome indicated 97% sequence identity (shown in Figure 3b). 

#### 2.3.2. BLAST and RetroTector^©^ Analysis

To identify the origin of the RD114-like sequence, a blastn search was performed using the sequence of the MDCK-RD114 PCR fragment to interrogate the *Canis lupus familiaris* (TAXID: 9615) database. Sequence hits with strong E-values ranging from 9e-23 to 2e-17 were found within several BAC clones (designated as A–L in Figure 2b and listed in Table 4). Since the PLEX-ID amplicon was identified as RD114 and the sequence shared strong sequence identity with several *Canis lupus familiaris* BAC clones, an alignment was done with RD114 complete retrovirus genome (Genbank ID AB705392.1) as the query and the identified *Canis lupus familiaris* BAC clones (A–L) as the subject entries (Figure 2b). Although PLEX-ID identified viral sequences belonging to RD114, the blastn alignment revealed three small regions (region 1, 2, and 3) with high alignment scores (80–200 and >200) to RD114 *gag* and *pol* (Figure 2b). Each of these BAC clones was located in different regions of chromosome 1 or in different chromosomes altogether. Since gammaretrovirus-like elements have been shown to exist in abundance throughout the *Canis lupus familiaris* genome [37], it is likely that the viral sequences detected by PLEX-ID belong to RD114-like ERVs. 

To determine the retroviral genome structure of the RD114-related ERVs within the *Canis lupus familiaris* genome, the identified BAC clones were analyzed using RetroTector^©^ [38], a free-share software designed to detect and characterize entire or fragmented ERVs in chromosomal DNA. Proviral motifs were found in at least one location within each BAC clone; the range spanned by the provirus is listed in column 5 of Table 4. In some cases, two proviral sequence chains (seq) were detected within a single BAC clone. For example, clones xx-73E6, and xx-219I19 each harbored two potential proviral regions; one provirus sequence chain shared similarity with RD114 (e.g., CfERV A and D) and the other sequence chain did not (e.g., CfERV M and N). It is likely proviruses that did not have homology to RD114 were related to other retroviruses [37].

Different RD114-like ERVs were found in several chromosomes including chromosome 1, 3, 8, 13, 25, and X. Although RetroTector^©^ detected many provirus motifs [38], many were small, incomplete, or segmented as indicated by the incomplete list of motifs shown in column 7 of Table 4. Some proviruses like those found within clone xx-73E6 and xx-35E2 appear to possess many motifs, but only a fraction of the motif is present. Thus, these ERVs are likely inactive as a result of genetic duplications, insertions, deletions, and/or frameshifts that have occurred during host duplication over millions of years. These variations are clearly depicted in the alignment shown in Figure 2b, where different degrees of RD114 similarity are found in certain regions within each BAC clone. 

Due to the variable degrees of similarity found between the proviral sequences, a phylogenetic analysis was conducted using nucleotide sequences from region 3 (Figure 2b), since this was common to all 12 canine ERVs including the PLEX-ID PCR fragment amplified from MDCK cells. The nucleotide span of region 3 within each BAC clone is defined in column 6 of Table 4. In addition, analogous *pol* regions of human T-lymphotropic virus 1 (HTLV-1) and ALV were used as distant delatretrovirus and alpharetrovirus homologs, respectively. We aligned the nucleotide sequences using both ClustalW and MUSCLE alignment options in MEGA 5.1 (Molecular Evolutionary Genetics Analysis program) [36] to ensure the results were robust and not dependent on the type of alignment used. The phylogeny was constructed using a maximum-likelihood analysis based on the Kimura 2-parameter model (Figure 3) [34]. Bootstrapping (1000 replicates) was used to support the confidence at each node [35]. The results suggest that these ERVs diverged from a gammaretrovirus, perhaps RD114, but have undergone significant genetic change as demonstrated by the divergence within this set of sequences (Figure 3a).

**Table 4:** Proviral regions within the *Canis familiaris* BAC clones detected by RetroTector.

Provirus ID	BAC Clone XX-(Chain)	Chr ^a^	Accession	RetroTector Provirus Location ^b^		Region 3 ^c^			Provirus Motifs Detected ^f^
						Range ^d^		% ^e^	
CfERV-A	73E6 (seq1)	1	AC184807.10	5394	14,041	9902	10,264	73	5' LTR, PBS, MA, CA, RT, IN, TM
CfERV-B	309C2 (seq1)	1	AC194581.5	143,113	150,196	146,057	146,419	73	5' LTR, PBS, MA, CA, RT, IN, TM
CfERV-C	412K16 (seq1)	1	AC191152.6	47,384	51,060	47,352	47,712	72	PBS, MA, CA, RT, IN
CfERV-D	219I19 (seq1)	3	AC186968.15	71,780	76,840	73,083	73,454	74	5' LTR, PBS, CA, NC, PR, IN, 3 'LTR
CfERV-E	231B7 (seq3)	8	AC194586.17	211,515	222,372	220,330	220,712	70	5' LTR, PBS, CA, NC, PR, RT, IN, TM, 3' LTR
CfERV-F	408G5 (seq1)	13	AC188926.6	167,789	173,976	170,817	171,150	68	5' LTR, PBS, CA, MA, NC, PR, TM
CfERV-G	239F12 (seq1)	25	AC186972.16	89,015	94,074	93,745	94,113	73	RT, IN
CfERV-H	146G22 (seq2)	X	AC186068.17	147,356	151,076	149,410	149,580	76	CA, NC, PR, IN
CfERV-I	35E2 (seq1)	X	AC187326.11	106,297	114,944	109,230	109,569	72	5' LTR, PBS, MA, CA, NC,PR, RT, IN, TM, 3' LTR
CfERV-J	306G8 (seq1)	X	AC189722.4	76,817	80,919	79,993	80,358	72	PBS, MA, CA, PR, IN
CfERV-K	494D7 (seq1)	14	AC189730.3	3268	8924	5573	5939	74	5' LTR, PBS, CA, NC, IN, TM
CfERV-L	224p2 and 282j14 (seq1)	12	AJ630363.1	113,754	118,869	115,518	115,882	73	PR, IN, TM
CfERV-M ^g^	73E6 (seq3)	1		207,305	216,077	n/a	n/a		5' LTR, PBS, MA, CA, NC, IN, TM, 3' LTR
CfERV-N ^g^	219I19 (seq3)	3		219,296	226,087	n/a	n/a		5' LTR, PBS, CA, NC, PR, RT, IN, 3' LTR
CfERV-O ^h^	35E2 (seq2)	X		106,282	115,165	n/a	n/a		5' LTR, PBS, MA, CA, NC, PR, RT, IN, TM, 3' LTR

^a^ Nucleotide location of provirus on the BAC clone; ^b^ Chromosomal location of BAC clone; ^c^ Region of RD114 homology in Figure 2b; ^d^ Nucleotide location of Region 3 on the BAC clone; ^e^ Percent sequence identity to RD114; ^f^ Long terminal repeat [LTR], primer binding site [PBS], matrix [MA], capsid [CA], nucleocapsid [NC], protease [PR], reverse transcriptase [RT], integrase [IN], transmembrane [TM]; ^g^ Proviral region had no similarity to RD114; ^h^ Proviral region was very similar to 35E2 seq1.

## 3. Experimental

### 3.1. Cell Lines

Cell lines were obtained from American Type Culture Collection (ATCC, Manassas, VA, USA) except as indicated. Trypsin (Gibco, Grand Island, NY, USA, cat. No. 25300-054) was used for passaging all adherent cell lines, except the insect cell lines, which were dislodged by scraping. Each cell line was passaged at least three times before the cells were harvested at the passage number indicated below and washed twice with 1× PBS (Quality Biologicals, Gaithersburg, MD, USA, cat. No. 114-058-101). After washing, 2 × 10^6^ cells were pelleted at 1200 rpm at 4 °C (Allegra™ 6KR centrifuge, Beckman Coulter, Danvers, MA, USA) and frozen at −80 °C for further nucleic acid extraction.

MDCK (Madin-Darby canine kidney, passage 64; ATCC, cat. No. CCL-34), HEK-293, (human embryonic kidney, passage 42; ATCC, cat. No. CRL-1573), CV-1, (African green monkey kidney, passage 39; ATCC, cat. No. CCL-70), HeLa (human cervical carcinoma, passage 100–112; ATCC cat. No. CCL-2), MRC-5 (human diploid fetal lung, passage 23; ATCC, cat. No. CCL-171), and VERO (African green monkey kidney, passage 127; ATCC, cat. No. CCL-81) were grown in Eagle’s minimal essential medium (EMEM, Mediatech, Manassas, VA, USA, cat. No. 15-010-CV) supplemented with 10% fetal bovine serum (FBS, heat-inactivated at 56 °C for 30 min; Hyclone, Logan, UT, USA, cat. No. SH30071.07) or 5% FBS in case of VERO, 100 U penicillin per mL and 100 µg streptomycin per mL (Quality Biological, cat. No. 120-095-721), 2 mM l-glutamine (Quality Biological, cat. No. 118-084-721), 1× nonessential amino acids (MEM-NEAA, Quality Biological, cat. No. 116-078-721), and 1 mM sodium pyruvate (Quality Biological, cat. No. 116-079-060). 293T/17 (human fetal kidney, passage 21; ATCC, cat. No. CRL-11268) and A549 (human lung carcinoma, passage 84; ATCC, cat. No. CCL-185) were grown in Dulbecco’s Modified Eagle Medium (DMEM; Gibco, Grand Island, NY, USA, cat. No. 11995-065) supplemented with 10% FBS, 100 U of penicillin per mL and 100 µg of streptomycin per mL, and 2 mM l-glutamine. Cf2Th (neonate canine thymus, passage 62; ATCC, cat. No. CRL-1430) were grown in DMEM, 20% FBS, 100 U of penicillin per ml and 100 µg of streptomycin per ml, 2 mM l-glutamine, and 1× MEM-NEAA. 

Raji (human lymphoblastoid lymphoma, FDA passage 5; ATCC, cat. No. CCL-86) was grown in RPMI 1640 (Quality Biological, cat. No. 112-024-101), 10% FBS, 100 U penicillin per mL and 100 µg streptomycin per mL, 2 mM l-glutamine, and 1× MEM-NEAA. A204 (human rhabdomyosarcoma, passage 84; ATCC, cat. No. HTB-62) was grown in RPMI 1640, 10% FBS, 100 U penicillin per mL and 100 µg streptomycin per mL, 2 mM l-glutamine, and 1× MEM-NEAA. 

Sf9 (*Spodoptera frugiperda* ovary, passage 25; ATCC, cat. No. CRL-1711) and High Five (*Trichopulsia ni* ovary, FDA passage 5; Invitrogen, Grand Island, NY, USA, cat. No. P/N51-4005) were grown in Grace’s Insect Medium (Invitrogen, cat. No. 11605-094), supplemented with 10% FBS (FBS, heat-inactivated at 56 °C for 30 min; certified for insect cells Hyclone, cat. No. SH30070.03), 100 U penicillin per mL and 100 µg streptomycin per mL, and 2 mM l-glutamine. Schneider’s Drosophilia line 2 (SL2) (*Drosophila melanogaster* embryo, passage 21; D.Mel.(2); ATCC, cat. No. CRL-1963) was grown in Schneider’s Drosophila Medium (Invitrogen, cat. No. 21720-024), supplemented with 10% FBS (certified for insect cells), 100 U penicillin per mL and 100 µg streptomycin per mL, and 2 mM l-glutamine. 

### 3.2. Preparation of XMRV and SFV-1 RNA Panels with and without Background Sf9 Total Nucleic Acids and RT-PCR Assays

XMRV and SFV-1 virus stocks were prepared using LNCaP and *Mus dunni* cells, respectively. The infectious titer for XMRV was 10^4.5^ TCID_50_ per mL [39] and for SFV-1 was 10^5.5^ TCID_50_ per mL. For both viruses, 10-fold serial dilutions (range 10^0^–10^−7^) were made from the original virus stock and the number of particles per µL was determined based upon RT activity using a modified STF-PERT assay (designated as two-step fluorescent-PERT; TSF-PERT). The number of particles in the original stock was determined as the average of the calculated particles at each dilution based upon a standard curve using HIV-1 RT enzyme (Worthington Biochemical Corporation Lakewood, NJ, USA, cat. No. LS05006; lot No. X1H2839), which was determined to have 262 pU of RT activity per particle [40]. The STF-PERT one-step assay [40] was modified into a two-step assay that no longer requires the use of AmpliWax^®^ PCR Gem 50, a product that has been discontinued. In addition, the TSF-PERT assay was designed as a partially automated process compatible with the Eppendorf epMotion 5070 robot (Eppendorf, Hauppauge, NY, USA, cat. No. 960000111). The RT master mix, standards, and samples were manually added to the 96-well plate as previously described [40] and the RT reaction was carried out using an Eppendorf MasterCycler ProS (Eppendorf, cat. No. 950030020). Once complete, the RT plate was placed on a 96-well cold Thermoblock (Eppendorf, cat. No. 960002083) and the PCR master mix was placed on a TMX 24 × SafeLock Thermorack (Eppendorf, cat. No. 960002070). The Eppendorf epMotion 5070 robot dispensed 25 µL of the PCR master mix into each well. Finally, the plate was sealed and processed for Taqman qPCR. The results indicated that XMRV had 3.12 × 10^5^ particles per µL and SFV-1 had 4.02 × 10^5^ particles per µL. 

Total viral RNA was extracted from each dilution using the QIAamp viral RNA mini kit (Qiagen, Valencia, CA, USA, cat. No. 52904) in combination with the RNase-free DNase I set (Qiagen, cat. No. 79254) according to the manufacturer’s instructions. Briefly, 200 µL of each dilution was processed as specified by the QIAamp viral RNA mini kit with an added on-column DNase I treatment. The RNA was eluted in 200 µL of DNase/RNase-free water. Concentration and purity were determined by using UV absorbance.

Viral RNA panels were made in the presence and absence of background Sf9 cell nucleic acids. Total cell nucleic acid was extracted from Sf9 cell pellets using the QIAamp^®^ MinElute^®^ Virus Spin Kit (Qiagen, cat. No. 57704). Briefly, cell pellets (2 × 10^6^ cells) were resuspended in 200 µL buffer and processed as specified in the QIAamp^®^ MinElute^®^ Virus Spin Kit followed by elution in 50 µL of DNase/RNase-free water. RNA panels (10^−2^–10^−6^) were initially made in a background of 10^6^ cell equivalents of Sf9 total nucleic acids or in water to create 10-fold and 100-fold dilutions for testing in a background of 10^5^ and 10^4^ cell equivalents of Sf9 total nucleic acid. Selected samples were tested in different assays. 

cDNA was synthesized using iScript cDNA synthesis kit (Bio-Rad, Hercules, CA, USA, cat. No. 170-8890) according to the manufacturer’s instructions. An XMRV gag fragment (500 bp) was amplified by a nested PCR assay using viral cDNA (2 µL) with an annealing temperature of 63 °C for the outer primers, as previously described [41]. An SFV-1 gag fragment (548 bp) fragment was also amplified by a nested PCR assay. The first amplification was done with primers SFVgagF1 (5'-AACCTAGGTGGAGAGCTGAAGG-3') and SFVgagR1 (5'-ATGGAGAGGGTAAGAACCATGGG-3') to generate a 947 bp fragment. This was followed by a second amplification with primers SFVgagF2 (5'-ATGATGCACTTTGGCAGCCATTGG-3') and SFVgagR2 (5'-ACCTGCTGAATGTTGATTCTGTGC-3') to generate a 578 bp fragment. Viral cDNA (2 µL) was used in a 50 µL reaction containing 1.5 U Taq DNA Polymerase (Roche Applied Science), 0.5 mM primers, 0.2 mM dNTP mix, and 1× PCR buffer with 1.5 mM MgCl_2_ (Roche Applied Science, Indianapolis, IN, USA). The PCR conditions used were: 94 °C for 3 min, (94 °C for 30 s, 55 °C for 1 min, 72 °C for 1 min) × 35 cycles, 72 °C for 10 min, and a 4 °C hold for infinity. In each case a control sample without reverse transcriptase (RT) was included. 

DNA fragments were visualized with ethidium bromide staining after running 10 µL of each PCR reaction on a 1.4% agarose gel. DNA sizes were determined using a 100 bp DNA ladder (New England Biolabs, Ipswich, MA, USA, cat. No. N3231L).

### 3.3. PCR/ESI-MS (PLEX-ID)

Cell pellets (2 × 10^6^ cells) were processed in our laboratory or were sent to Athogen for processing [20]. In addition, total nucleic acids were extracted in our laboratory from PBS, ultra-pure dH20, complete medium (DMEM supplemented with 10% FBS (Hyclone, Logan, UT, USA, cat. No. SH30071.07, lot. No. ATE32066), 100 U of penicillin per ml and 100 µg of streptomycin per mL, l-glutamine and 1× MEM-NEAA), and trypsin (Gibco, Grand Island, NY, USA, cat. No. 25300-054, lot No 917992). Briefly, cell pellets were resuspended in 200 µL AVE buffer or 200 µL of liquid sample were processed using the QIAamp^®^ MinElute^®^ Virus Spin Kit (Qiagen, cat. No. 57704) as described by the manufacturer. Total nucleic acids were eluted in 200 µL of DNAse/RNAse-free water. All samples were analyzed using PLEX-ID Biopharma Viral Assay Kits (Ibis BioSciences, Abbott, Carlsbad, CA, USA): Bovine origin virus, Porcine origin virus, CHO cell virus, and Other virus, either in our laboratory using an on-site PLEX-ID or sent to Athogen for testing. In all cases, a one-step 50 µL RT-PCR reaction containing 5 µL of total nucleic acids extract was performed as previously described [42]. All PCRs were conducted in a 96-well plate format using an Eppendorf Mastercycler under conditions previously described [14,42]. Samples were processed by an automated carousel contained with the PLEX-ID machine, where PCR samples were desalted and injected into the ESI-TOF mass spectrometer. Once the signal was processed, genomic signatures were generated by Ibis software and compared against Athogen’s curated database. 

### 3.4. LLMDA and Virohip

RNA, DNA, and total nucleic acid samples were sent to Lawrence Livermore National Laboratory (LLNL, Livermore, CA, USA) and University of California, San Francisco (UCSF, San Francisco, CA, USA) to be analyzed by LLMDA [4] and ViroChip v.5 [2,21,22], respectively. Briefly, each RNA sample (10 µL for the LLMDA and 11 µL for the Virochip) was reverse-transcribed to cDNA using random primers and PCR-amplified prior to labeling with Cy3 fluorescent dye and hybridization to the arrays [2,4].

An updated LLMDA v.5 that was designed to detect around 5500 microbial species, which were sequenced through December 2011, was used for our studies. This study employed the 12-plex 135 K format of this array, which is restricted to pathogens associated with vertebrate infection, and includes 1856 viral, 1398 bacterial, 123 archaean, 48 fungal, and 94 protozoan species. In the LLMDA v.5 [4], in order to expand the abilities to detect these viruses from cell lines probes were also designed to detect additional mammalian endogenous retroviruses and some insect viruses. These included: 6 eukaryotic sequences (*Drosophila simulans* clone F pop-variant Chicharo (Portugal) retrotransposon tirant envelope protein (*env*) gene partial cds (Genbank ID JN786103.1), *Drosophila melanogaster* copia-like element 17.6 (Genbank ID X01472.1), *Drosophila melanogaster* Idefix retroelement: *gag pol* and *env* genes partial (Genbank ID, AJ009736.1), *Drosophila melanogaster* transposable element 297 (Genbank ID, X03431.1), *Drosophila melanogaster* clone 4.1 gypsy retrotransposon MDG4 complete sequence (Genbank ID, DQ887187.1), *Homo sapiens* gypsy retrotransposon integrase 1 (GIN1) mRNA (Genbank ID, NM_017676.2); 9 partial sequences of *Spodoptera frugiperda* insect viruses [33], 1 baculovirus (*Autographa californica* nuclear polyhedrosis virus (AcMNPV) mutant FP-D with incorporated *Trichoplusia ni* retrotransposon TED (Tn368) and three open reading frames (Genbank ID M32662.1); and 3 endogenous retroviruses (*Odocoileus hemionus* endogenous virus Cervid endogenous retrovirus CrERVg complete genome (Genbank ID JN592050.1), Simian retrovirus 1 isolate SRV_Vero_Assembled gag pseudogene partial sequence; pol protein (pol) gene partial cds; and env pseudogene complete sequence (Genbank ID HM143845.1), and *Python molurus* endogenous retrovirus Gag-Pro-Pol protein and Env genes complete cds (Genbank ID AF500296.1). 

The ViroChip v.5 used in this study was an 8-plex 60K format and was updated to include 60,000 probes representing complete and partial sequences of all viral genomes in GenBank as of December 2010 [21,22]. 

### 3.5. PCR Assay for RD114-Like Virus and Nucleotide Sequencing Analysis

A positive hit for RD114 by PLEX-ID was verified using a nested PCR assay and nucleotide sequencing. PCR primers were developed based upon the PLEX-ID broad-range forward and reverse primer set 3417 sequences (kindly provided by Rangarajan Sampath, Ibis BioSciences, Abbott, Carlsbad, CA): outer primers were 3417F (5'-TGGGGATTGATTGGAAATTAC-3') and 3417R (5'-TGTTCTATTCATTCTTTCTAC-3'); an inner primer set was designed: 3417F2 (5'-TATTCATTCTTTCTACCTGTCGTG-3') and 3417R2 (5'-ATTGATTGGAAATTACATTGTGC-3'). Total nucleic acids (5 µL) from MDCK cells were amplified in a PCR reaction of total volume 50 µL containing 1.5 U Taq DNA Polymerase (Roche Applied Science, cat. No. 11647687001), 0.5 mM primers, 0.2 mM dNTP mix, and 1× PCR buffer with 1.5 mM MgCl_2 _(Roche Applied Science, cat No 11647687001). The PCR conditions used were: 95 °C for 5 min (95 °C for 30 s, 55 °C for 1 min, 72 °C for 1 min) × 35 cycles, 72 °C for 10 min, and a 4 °C hold for infinity. A 74 bp fragment was amplified after the first round of PCR using the outer primers and a 64 bp fragment was amplified by a second amplification with the inner primers. 

DNA fragments were visualized by ethidium bromide staining after running 20 µL of the PCR reaction on a 4% NuSieve GTG gel (Lonza, Rockland, ME, USA, cat. No. 50081). DNA sizes were determined using ΦX174 DNA/*Bsu*R I (*Hae* III) marker (Fermentas, Inc., Glen Burnie, MD, USA, cat. No. SM0253). DNA fragments were purified from the gel fragment by using Zymoclean™ Gel DNA Recovery Kit (Zymo Research Corp., Orange, CA, USA, cat. No. D4001) according to the manufacturer’s instructions.

The purified PCR fragments were cloned into pGEM-T Easy Vector (Promega, Fitchburg, WI, USA, cat. No. A1380) as per the manufacturer’s instructions and transformed into JM109 high efficiency competent cells. Several colonies were picked and were grown in S.O.C. medium (Invitrogen, Carlsbad, CA, USA, cat. No. 15544-034) at 37 °C overnight. Plasmid preps were made using QIAprep^®^ Spin Miniprep kit (Qiagen, cat. No. 27106) and sequenced with the T7 primer using Big Dye v3.1 (Applied Biosystems, Foster City, CA, USA, cat. No. 4337455). Nucleotide sequences were generated on an ABI 3130*xl* Genetic Analyzer and sequence analysis was done using Vector NTI (Invitrogen). The identity of the PCR fragments amplified from the first and second PCR amplifications was confirmed by nucleotide sequencing; since both had similar overlapping sequences the larger 74 bp sequence was used for subsequent analysis.

Bioinformatic and phylogentic analysis were done using BLAST (National Center for Biotechnology Information, National Library of Medicine, NIH, Bethesda, MD, USA), CLC Genomics workbench (CLC Bio., Cambridge, MA, USA), RetroTector^©^ (Uppsala Universitet, Uppsala, Sweden), and MEGA 5.1 (Molecular Evolutionary Genetics Analysis, Tempe, AZ, USA). 

## 4. Conclusions

The results showed that the LOD of PLEX-ID for detection of XMRV was equivalent to RT-PCR in the absence of background nucleic acids, and greater by one log in the presence of 10^5^ cell equivalents of Sf9 total nucleic acids (Table 1). However, SFV-1 could not be detected by the PLEX-ID due to the absence of relevant primers. LLMDA was a log more sensitive for detection of both XMRV and SFV-1 as compared to PLEX-ID and RT-PCR, but LLMDA could not detect these viruses in a background of 10^4^ cell equivalents of Sf9 total nucleic acids. Selected samples of the XMRV (RNA dilutions 10^−6^–10^−7^) and SFV-1 RNA panels (RNA dilutions 10^−5^–10^−6^) were also tested with the ViroChip. In the absence of Sf9 total nucleic acids, the XMRV 10^−6^ RNA dilution sample was detected by the ViroChip, whereas, the SFV-1 RNA dilution 10^−5^ was not detected. In addition to the LOD studies, we used the new virus detection methods to analyze some vaccine-related cell substrates for the potential presence of novel viruses. Total nucleic acids were used for the detection of both DNA and RNA viruses. Analysis of cell lines by PLEX-ID and virus microarrays indicated the presence of various retrovirus-related sequences. However, the origin of these sequences could be attributed to the presence of cellular DNA containing endogenous retroviral sequences or viral transcripts that may be expressed constitutively from some endogenous retroviral DNAs (proviruses) [43]. However, this situation is applicable to all nucleic acid-based assays that can detect retroviruses. To assess the origin and nature of the retroviral sequences a follow up strategy using PCR, sequencing, and bioinformatics was developed. The analysis revealed that the unexpected RD114 sequences detected by PLEX-ID in canine cell lines were associated with defective endogenous gammaretroviruses. Similarly, blast analysis and amplicon base count comparisons also revealed that endogenous retrovirus sequences were responsible for positive hits within the alpharetrovirus and betaretrovirus families detected in human cell lines.

Our analysis further showed that different viruses were detected by using different technologies. These results emphasize the need to use a combination strategy for virus detection in evaluating the safety of biologicals or screening patient samples. It was noted that some expected virus sequences that were known to be present as fragments in some cells lines were not detected by any of the methods: e.g., adenovirus in HEK293 cells and 293T/17 cells, and HPV in HeLa cells. This may be due to the absence of primers for the viral specific regions in the assays. These results also indicate the need for further updates of the PLEX-ID panels and virus arrays to enhance detection of known and novel viruses.
