# Correction: Taliaferro, L. *et al.* Evaluation of the Broad-Range PCR-Electrospray Ionization Mass Spectrometry (PCR/ESI-MS) System and Virus Microarrays for Virus Detection. *Viruses* 2014, *6*, 1876-1896

**DOI:** 10.3390/v6114664

**Published:** 2014-11-24

**Authors:** Lanyn P. Taliaferro, Teresa A. Galvin, Hailun Ma, Syed Shaheduzzaman, Dhanya K. Williams, Dustin R. Glasner, Arifa S. Khan

**Affiliations:** Laboratory of Retroviruses, Division of Viral Products, Center for Biologics Evaluation and Research, U.S. Food and Drug Administration, 8800 Rockville Pike, HFM-454, Bethesda, MD 20892, USA; E-Mails: lanyn.taliaferro@fda.hhs.gov (L.P.T.); teresa.galvin@fda.hhs.gov (T.A.G.); hailun.ma@fda.hhs.gov (H.M.); syed.shaheduzzaman@fda.hhs.gov (S.S.); dhanya.williams@fda.hhs.gov (D.K.W.)

We have noted some inaccuracies in Table 1 and Table 2 of our article “Evaluation of the Broad-Range PCR-Electrospray Ionization Mass Spectrometry (PCR/ESI-MS) System and Virus Microarrays for Virus Detection” (*Viruses* 2014, *6*, 1876–1896) [1].

In Table 1, the heading “LLMDA” was omitted and needs to be added to correctly align the results obtained using the LLMDA and the ViroChip with their corresponding results. Additionally, the 10^−7^ RNA dilution sample needs to be corrected to indicate that it was not tested (nt) in the presence of 10^5^ cell equivalents of Sf9 total nucleic acids (+10^5^) by the PLEX-ID. 

In Table 2, the results for LLMDA need to be corrected to indicate that 10^−4^ RNA dilution was tested and found to be negative, whereas the 10^−6^ RNA dilution was not tested (nt).

Below you will find the corrected Table 1 and Table 2 for our recently published article [1]. We apologize to the readers of *Viruses* for any inconvenience this may have caused.

**Table 1:** Limit of detection using XMRV RNA panel.

Sample			PLEX-ID		LLMDA		Virochip	RT-PCR ^a^	
RNA Dilution ^b^	TCID_50_/mL	Particles/µL ^c^	(−)Sf9	+10^5^	(−)Sf9	+10^5^	(−)Sf9	(−)Sf9	+10^5^
10^−3^	10^1.5^	312	+	+	+	nt ^d^	nt	+	+
10^−4^	10^0.5^	31.2	+	+	+	nt	+	+	+
10^−5^	10^−0.5^	3.12	+	+	+	-	+	+	-
10^−6^	10^−1.5^	0.312	-	-	+	-	+	-	-
10^−7^	10^−2.5^	0.0312	-	nt	-	nt	-	-	-

^a^ Results from nested PCR with XMRV *gag* primers; ^b^ Viral RNA dilution series were spiked into RNase/DNase free water [(−)Sf9] or present (+) in 10^5^ or 10^4^ cell equivalents of Sf9 total nucleic acids; ^c^ Calculated based upon RT activity determined by TSF-PERT assay; ^d^ Not tested.

**Table 2:** Limit of detection using SFV-1 RNA panel.

Sample			PLEX-ID	LLMDA		RT-PCR ^a^		
RNA Dilution ^b^	TCID_50_/mL	Particles/µL ^c^	(−)Sf9	(−)Sf9	+10^4^	(−)Sf9	+10^5^	+10^4^
10^−3^	10^2.5^	402	nt ^d^	nt	nt	+	+	nt
10^−4^	10^1.5^	40.2	-	+	-	+	-	+
10^−5^	10^0.5^	4.02	-	+	-	-	-	-
10^−6^	10^−0.5^	0.402	-	-	nt	-	-	nt

^a^ Results from nested PCR with SFV-1 *gag* primers; ^b^ Viral RNA dilution series were spiked into RNase/DNase free water [(−)Sf9] or present (+) in 10^5^ or 10^4^ cell equivalents of Sf9 total nucleic acids; ^c^ Calculated based upon RT activity determined by TSF-PERT assay; ^d^ Not tested.
